# Synthesis of Dendronized Poly(l-Glutamate) via Azide-Alkyne Click Chemistry

**DOI:** 10.3390/ma9040242

**Published:** 2016-03-29

**Authors:** Peter Perdih, Andrej Kržan, Ema Žagar

**Affiliations:** Laboratory for Polymer Chemistry and Technology, National Institute of Chemistry, Hajdrihova 19, SI-1000 Ljubljana, Slovenia; peter.perdih@helios.si (P.P.); andrej.krzan@ki.si (A.K.)

**Keywords:** dendronized polymer, poly(l-glutamate), bis-MPA, l-lysine, “click” reaction

## Abstract

Poly(l-glutamate) (PGlu) was modified with a second-generation dendron to obtain the dendronized polyglutamate, P(Glu-D). Synthesized P(Glu-D) exhibited a degree of polymerization (DP_n_) of 46 and a 43% degree of dendronization. Perfect agreement was found between the P(Glu-D) expected structure and the results of nuclear magnetic resonance spectroscopy (NMR) and size-exclusion chromatography coupled to a multi-angle light-scattering detector (SEC-MALS) analysis. The PGlu precursor was modified by coupling with a bifunctional building block (N_3_-Pr-NH_2_) in the presence of 4-(4,6-dimethoxy-1,3,5-triazin-2-yl)-4-methylmorpholinium chloride (DMTMM) coupling reagent. The second-generation polyamide dendron was prepared by a stepwise procedure involving the coupling of propargylamine to the l-lysine carboxyl group, followed by attaching the protected 2,2-bis(methylol)propionic acid (bis-MPA) building block to the l-lysine amino groups. The hydroxyl groups of the resulting second-generation dendron were quantitatively deprotected under mild acidic conditions. The deprotected dendron with an acetylene focal group was coupled to the pendant azide groups of the modified linear copolypeptide, P(Glu-N_3_), in a Cu(I) catalyzed azide-alkyne cycloaddition reaction to form a 1,4-disubstituted triazole. The dendronization reaction proceeded quantitatively in 48 hours in aqueous medium as confirmed by ^1^H NMR and Fourier transform infrared spectroscopy (FT-IR) spectroscopy.

## 1. Introduction

Dendronized polymers consist of dendrons attached to a linear polymer backbone [1,2,3,4]. With the growing dendron generation number the dendronized polymers tend to change their conformation from a random coil or helical conformation to a more extended conformation due to the steric crowding of the bulky dendritic wedges [2,5]. However, the extension and flexibility of dendronized polymers depend beside on the dendron generation also on the spacer type between the backbone and the dendron, the graft density, and the backbone polymer itself [2]. The impact of dendron generation [6] and the presence of charged peripheral groups [7,8] to the structure of dendronized polymers was investigated thoroughly by Schlüter’s group. Dendronized polymers possess a large number of peripheral functional groups due to their branched structure. High functionality, together with the distinctive size and shape, make the dendronized polymers very interesting for potential application in various fields such as biomedicine [9,10,11,12], recognition of metal cations [13,14,15], biocatalysis [16], bacteria detection [17], and to prepare self-assembled ordered structures [2,18].

There are three general synthetic approaches for the synthesis of dendronized polymers [2,19]: (i) *graft-to*, where the preformed dendrons are coupled to the linear backbone, (ii) *graft-from*, where the dendrons are prepared divergently from the linear backbone, and (iii) *macromonomer approach*, where the dendron bearing a polymerizable focal group is polymerized.

To efficiently perform the coupling of a preformed dendron to the linear polymer when following the graft-to approach, highly efficient reactions are preferred such as Cu(I)-catalyzed azide-alkyne cycloaddition (CuAAC) [20], Diels-Alder cycloadition [21] and thiol-ene click reaction [22]. A decade ago the applicability of the CuAAC reaction for this end was investigated by Fréchet's group and others [23,24,25,26]. Dendronized polymers were, furthermore, prepared by applying anthracene-maleimide Diels-Alder cycloaddition either alone [27], or in combination with CuAAC [24]. In the latter case, polyaryl ether dendrons were coupled to the modified polystyrene linear polymer via CuAAC followed by Diels-Alder coupling of 2,2-bis(methylol)propionic acid (bis-MPA) dendrons. Diels-Alder cycloaddition was also applied for the preparation of a set of glyco-dendronized poly(l-lysines), where the maleimide-functionalyzed poly(l-lysine) reacted with the glyco-dendrons bearing an alkyne focal group [17]. The thiol-ene click reaction was applied in the coupling of bis-MPA dendrons bearing a thiol focal group to the alkene-modified polymethacrylate [28]. Furthermore, CuAAC was applied for the preparation of a series of dendritic macromonomers, which were then polymerized by a furan–maleimide Diels-Alder cycloadition reaction, thus following the macromonomer approach [29,30].

In addition to the three general synthetic approaches (*graft-to*, *graft-from* and *macromonomer approach*), a combined approach was applied by Schlüter *et al.* [31], Fréchet *et al.* [32] and Malmström *et al.* [33] more than a decade ago where low-generation dendronized polymers were prepared by the *macromonomer approach* followed by growing the dendron generation using the *graft-from* approach. Schlüter’s group further developed the combined synthetic strategy—“*n* + 2” approach, where a second-generation dendron was coupled to a pre-formed dendronized polymer [34]. This strategy was recently applied to prepare a high molar mass, eighth-generation dendronized polymer [35].

To avoid the described demanding synthetic approaches for the preparation of dendronized polymers the *supramolecular approach*, exploring non-covalent interactions like hydrogen bonding, metal coordination, π–π interactions, ionic interactions, and host-guest inclusions, was also designed [2,18]. Dendronized supramolecular polymers are typically formed either (i) from dendrons bearing supramolecular motifs as their focal groups, thus resembling the *macromonomer approach*; or (ii) through non-covalent interactions between a linear polymer chain and the dendrons, similar to the *graft-to* approach.

Dendronized polymers have been extensively investigated for potential application in biomedicine. Dendronized poly(4-hydroxystyrene) bearing bis-MPA dendrons was tested *in vitro* and *in vivo* and was found to be a suitable candidate for drug delivery applications [10]. Synthesis of bis-MPA-based dendronized polymers and other dendritic materials has been reviewed in detail by Carlmark *et al.* [36,37]. Poly-l-lysine dendrimers were successfully investigated for DNA delivery applications more than a decade ago [38,39]. In addition to that, a comparative study of DNA delivery with linear, hyperbranched, and dendritic poly-l-lysine was undertaken [40] to prove the importance of poly-l-lysine architecture variation for biomedical applications. The synthesis of l-lysine dendrons was described for linear-dendritic hybrids [41,42,43,44], dendrimers [38,45,46,47,48,49], and dendronized polymers [1,50,51]. The l-lysine dendrons were typically prepared using urethane or trifluoroacetyl protection/deprotection chemistry and the amide linkages were prepared by carboxyl group activation followed by reaction with deprotected peripheral amine groups.

Poly(α-amino acids) or polypeptides were suggested as suitable backbones for dendronized polymer synthesis due to the ease of preparation and the presence of functional groups in the resulting backbone. In addition, they are bio-based and reported to be biocompatible [5]. Poly(l-glutamate) (PGlu) dendronized with polyaryl ether dendrons were found to self-assemble into highly-ordered liquid crystalline nanostructures [52]. The dendronized PGlu was prepared by coupling diazo-functionalized dendrons to the carboxylic side groups of PGlu using the *graft-to* approach [52]. Poly(l-lysine) dendronized with bis-MPA dendrons were investigated for generation-dependent conformation change [5]. The dendronized polymer was prepared by applying the *graft-from* approach where bis-MPA monomers were coupled to the polymer using the acetonide-protected bis-MPA anhydride [5]. 

Next, poly(l-lysine) dendronized with glycosilated dendrons was applied for *Escherichia coli* bacteria detection [17]. The glycosylated dendrons with alkyne focal groups were coupled to the maleimide functionalized poly(l-lysine) under ultra-violet (UV)-irradiation, thus applying the *graft-to* approach [17]. Furthermore, a set of poly(l-prolines) was dendronized with oligoethylene glycol dendrons [25]. The thermoresponsive dendronized polypeptides were prepared via *graft-to* approach by applying CuAAC. The oligoethylene glycol dendrons with alkyne focal group were coupled to the azide-functionalyzed l-proline repeat units of the polypeptide backbone [25]. Finally, a library of poly(cystine-co-l-lysine) polypeptides dendronized with different functionalized poly(l-lysine) dendrons was investigated for safe and effective small, interfering RNA delivery [53]. The linear backbone poly(cystine-co-l-lysine) was prepared from di-Boc-protected cystine and l-lysine ethyl esters using 1-ethyl-3-(3-dimethylaminopropyl)carbodiimide (EDC) coupling reagent and hydroxybenzotriazole (HOBt). Then, the dendrons were grown from the linear backbone following the *graft-from* approach by using (benzotriazol-1-yloxy)tris(dimethylamino)phosphonium hexafluorophosphate (BOP) coupling reagent [53].

The aim of our study was to design, prepare and characterize a dendronized poly(l-glutamate) (P(Glu-D)) consisting of a negatively charged polypeptide backbone dendronized by a second-generation dendron prepared from bis-MPA and amino acid l-lysine. The backbone was prepared by ring-opening polymerization (ROP) of γ-benzyl-l-glutamate *N*-carboxyanhydride (BGlu NCA) followed by deprotection and modification in a well-defined manner to incorporate the pendant azide functional groups to the polypeptide backbone. The second-generation dendron was designed to have an acetylene focal group and hydroxyl peripheral functional groups. *Graft-to* synthetic approach was then applied for the preparation of the dendronized polypeptide P(Glu-D). 1D and 2D nuclear magnetic resonance spectroscopy (NMR), size-exclusion chromatography coupled to a multi-angle light-scattering detector (SEC-MALS), and matrix-assisted laser desorption/ionization time-of-flight mass spectrometry (MALDI-TOF MS) techniques were applied for a detailed characterization of the resulting dendronized polypeptide to confirm the proposed structure of P(Glu-D).

## 2. Materials and Methods

### 2.1. Materials

All the reagents and solvents were used as received. Tetrahydrofuran (THF) (p.a.), *n*-hexane (p.a.), diethyl ether (p.a.), dichlorometane (p.a.), acetonitrile (p.a.), acetone (p.a.), NaOH (>99%) and NaHCO_3_ (>99.0%) were obtained from Merck, Darmstadt, Germany. Sodium (+)-l-ascorbate (>98%) was obtained from Sigma, Darmstadt, Germany. Triphosgene (>99.0%), *n*-hexylamine (99%), propargylamine (98%), 3-bromo-propanamine hydrobromide, 1-Hydroxybenzotriazole (HOBt) hydrate (97%), Amberlyst^®^ A21, and Boc-Lys(Boc)-OSu (97.0%) were obtained from Aldrich, St. Louis, MO, USA. 2,2-Bis(hydroxymethyl)propionic acid (Bis-MPA) (98%), 2,2-dimetoxypropane (98%), p-toluenesulfonic acid (PTSA) monohydrate (95.5%), Na_2_CO_3_ (>99.8%), Na_2_SO_4_ (anhydrous, >99.0%), THF (>99.9%, anhydrous), N,N-dimethylformamide (DMF) (anhydrous, 99.8%), trifluoroacetic acid (TFA) (99%), NaN_3_ (>99.5%), triethylamine (>99%), MSA (>99.5%), ammonia solution (2.0 M in ethanol), and anisole (99%) were obtained from Sigma-Aldrich, St. Louis, MO, USA. NaCl (Ph Eur), NaHSO_4_ (95%) were obtained from Fluka, Buchs, Switzerland. γ-benzyl-l-glutamate (BGlu) (>99%), 4-(4,6-Dimethoxy-1,3,5-triazin-2-yl)-4-methylmorpholinium chloride (DMTMM) (99+%), CuSO_4_·5H_2_O (99+%), and ethylenediaminetetraacetic acid (EDTA) (99%) were obtained from Acros Organics, Geel, Belgium. EDC (>98.0%) was obtained from TCI, Zwijndrecht, Belgium.

### 2.2. Methods

#### 2.2.1. NMR

^1^H, ^13^C, ^1^H-^1^H correlation spectroscopy (COSY), and ^1^H-^13^C gradient heteronuclear single quantum coherence adiabatic version (gHSQCad) spectra were recorded in DMSO-*d*_6_ or in D_2_O on a Varian Unity Inova 300 instrument (Oxford, UK) in the pulse Fourier-transform mode with a relaxation delay of 5 s, and an acquisition time of 3 s. Tetramethylsilane (TMS, δ = 0) and 3-trimethylsilyl-2,2′,3,3′-*d*_4_-propanoic acid sodium salt (TMSPA, δ = 0) were used as internal chemical-shift standards in DMSO-*d*_6_ and D_2_O, respectively.

#### 2.2.2. SEC-MALS

Size-exclusion chromatography coupled to a multi-angle light-scattering detector (SEC-MALS) measurements were performed using an isocratic pump with an online vacuum degasser and an autosampler (Agilent 1260 type, Agilent Technologies, Santa Clara, CA, USA). The separations of samples were carried out on a PolarGel-L column (Agilent Technologies, molar mass range: up to 30 kDa). Two different eluents were used: (i) 0.1 M sodium nitrate (NaNO_3_) solution with sodium azide (NaN_3_) (0.02% *w*/*v*) (both from Sigma-Aldrich), prepared with MiliQ water (18.2 MΩ/cm) at pH 10 for PGlu, P(Glu-N_3_) and P(Glu-D) samples, and (ii) dimethylacetamide (DMAc) with 0.05 M lithium bromide (LiBr) (both from Sigma-Aldrich) for PBGlu sample. The detection was done through a system of successively online connected detectors: a multi-angle light-scattering DAWN-HELEOS detector (MALS with 18 angles), operating at a wavelength of 658 nm, and an interferometric refractive index (RI) detector Optilab rEX (both instruments from Wyatt Technology Corporation, Santa Barbara, CA, USA), operating at the same wavelength as the MALS detector. The nominal eluent flow rate was 1.0 mL/min. The mass of the samples injected onto the column was typically 1 × 10^−4^ g, whereas the solution concentration was 1 × 10^−3^ g/mL. The determination of absolute Mw and the calculation of Mn values from MALS detector require a sample-specific refractive-index increment (d*n*/d*c*), which was determined from the RI response assuming 100% of sample mass recovery from the column. For the data acquisition and evaluation Astra 5.3.4 software (Wyatt Technology Corporation) was utilized.

#### 2.2.3. MALDI-TOF MS

The MALDI-TOF MS measurements were performed with a Bruker UltrafleXtreme MALDI-TOF mass spectrometer (Bruker Daltonik, Bremen, Germany) equipped with a 337 nm nitrogen laser, capable of executing reflector mode analysis. The reflector positive ion mode was used for acquiring the mass spectra of PBGlu, PGlu, and P(Glu-N_3_). The dried-droplet method was used to spot the samples on a MALDI plate. 

The solutions of PBGlu (2 mg/mL), super DHB (a 9:1 mixture of 2,5-dihydroxybenzoic acid (DHB) and 2-hydroxy-5-methoxybenzoic acid) (20 mg/mL), and potassium trifluoroacetate KTFA (5 mM) in THF were mixed in a volume ratio of 1:10:1. PGlu and P(Glu-N_3_) were dissolved in water (2 mg/mL) and mixed with a solution of super DHB (20 mg/mL) in 30/70 (*v*/*v*) mixture of acetonitrile/aqueous 0.1% TFA in a volume ratio of 1:10. In all cases 0.5 µL of the mixture was deposited on a MALDI target and allowed to dry on air.

The mass spectra were acquired by summing spectra from 500 selected laser shots. The calibration was made externally with a Peptide calibration standard II and Protein Calibration Standard I (Bruker Daltonik). The data was processed with Bruker FlexAnalysis 3.3.80 software.

#### 2.2.4. FT-IR

Fourier-transform infrared spectroscopy (FT-IR) spectra were recorded on a Perkin–Elmer Spectrum One instrument (Perkin-Elmer, Inc., Waltham, MA, USA). Sixteen scans were recorded at resolution of 4 cm^−1^ in the range of 400–4000 cm^−1^ using pressed KBr pellets.

## 3. Results and Discussion

### 3.1. Synthesis of the Second-Generation Dendron (D) with Acetylene Focal Group

In our effort to prepare a dendronized polypeptide copolymer following a “*graft-to*” synthetic approach a polyamide dendron was designed bearing a propargyl focal group and peripheral hydroxyl functional groups. The propargyl functionality allows effective coupling of a pre-formed dendron to the azide functionalized polyglutamate backbone under mild conditions by applying the CuAAC reaction.

**Figure 1:**
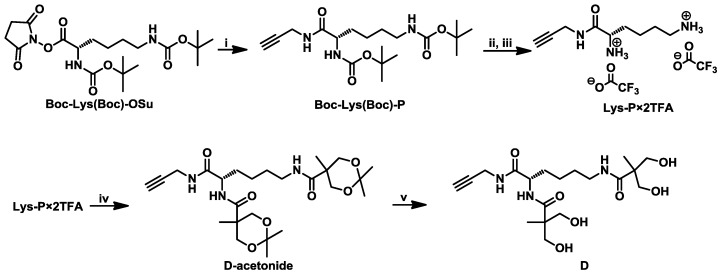
Reaction scheme for the preparation of dendron D. (**i**) Propargylamine, triethylamine, dichloromethane, r.t.; (**ii**) TFA:dichloromethane (1:1), r.t.; (**iii**) Amberlyst^®^ A21, acetonitrile; (**iv**) bis-MPA acetonide, triethylamine, EDC, HOBt, dichlorometane, 0 °C then r.t.; and (**v**) TFA:acetonitrile (1:20), r.t.

The dendron (Figure 1, Supplemental Materials) was prepared from propargylamine, l-lysine and bis-MPA building blocks, with the alkyne group of propargylamine moiety as the focal group, l-lysine as an amino acid building block, known for its application in synthesis of dendritic polymers [1,38,41,42,43,49,50], while bis-MPA served as a non-toxic and biocompatible neutral building block [10]. The dendron synthesis involved coupling of propargylamine to the l-lysine carboxylic group, while bis-MPA moiety was later coupled to the deprotected α- and ε-amino groups of the l-lysine propargylamide (Lys-P).

The synthesis of propargylamides and alkynyl amides of varius α-l-amino acids was already reported by Masuda’s and Metzler-Nolte’s groups [54,55,56,57,58] by applying either isobutyl chloroformate [55,57,58], EDC [56] or DMTMM [54] as the coupling agents. We synthesized Boc-Lys(Boc)-P from the commercially available activated Boc-Lys(Boc)-OSu precursor which was reacted with propargylamine in dichloromethane in the presence of triethylamine as a base. The product was purified by a simple aqueous work-up. The ^1^H NMR spectrum of Boc-Lys(Boc)-P (Figure S1A) shows signals assigned to the l-lysine and Boc protective groups as well as the signals belonging to the amide proton (signal *a*, 8.22 ppm) and terminal acetylene proton *1* at 3.08 ppm. The CH_2_ proton signal in position *3* (at 3.84 ppm) was overlapped with the l-lysine proton signal *5*. Signals *1* and *3* indicated the presence of the propargylamine moiety in the product while the signal marked with a clearly demonstrated that propargylamine is coupled to the amino acid by an amide bond.

In the ^13^C NMR spectrum of Boc-Lys(Boc)-P (Figure S1B) the carbon signals *1*, *2*, and *3* are present at 77.8, 81.0, and 27.8 ppm, respectively. The presence of the amide bond is indicated by the carbon signal *4* at 171.9 ppm that is at much higher chemical shift than the urethane carbons *10* and *10′*.

Boc protective groups were removed from Boc-Lys(Boc)-P by applying TFA in dichloromethane (Figure 1). The product, Lys-P × 2TFA was isolated as a TFA salt. In the ^1^H NMR spectrum of Lys-P × 2TFA (Figure S1C) a broad signal *b* was observed at 7.69 ppm, which was assigned to the protonated amino groups of the lysine moiety. The urethane signals at 6.4 and 6.7 ppm were absent as well as Boc signals (at 1.37 ppm) indicating complete deprotection. In the ^13^C NMR spectrum of Lys-P × 2TFA (Figure S1D) the Boc group signals were absent, however, the signals assigned to the TFA anion carbons at 115 and 158 ppm were observed.

Bis-MPA-acetonide was prepared by reacting bis-MPA with 2,2-dimethoxypropane [59]. Afterwards, bis-MPA-acetonide was coupled to the Lys-P×2TFA by amide bond formation (Figure 1). Lys-P × 2TFA was dissolved in dichloromethane, neutralized with triethylamine, and chilled on ice. Then, bis-MPA-acetonide, HOBt, and EDC were added and the reaction mixture was stirred for 20 h and allowed to warm up to room temperature. The product, d-acetonide, was purified by aqueous work-up, followed by flash-column chromatography. d-acetonide was characterized by 1D and 2D NMR experiments (Figure S2).

In the ^1^H NMR spectrum of d-acetonide, the amide protons *b* (doublet) and *c* (triplet) were observed in addition to the amide proton a (Figure S2A). In addition to the signals assigned to the lysine and propargylamine moieties, the bis-MPA signals *12*, *12′*, *13*, and *13′* together with acetonide protective group signals *15* and *15′* were observed. The ^13^C NMR spectrum of d-acetonide is rather complex in the region between 17 and 32 ppm (Figure S2B). However, the presence of bis-MPA is indicated in the ^13^C NMR spectrum by the signals *11*, *12*, and *13* at 40, 18, and 65 ppm while the carbonyl signals *10* and *10′* at 173 ppm indicate the amide bond formation between the lysine and bis-MPA moieties. Additionally, the signals *14* and *15*, which are characteristic of the acetonide protective groups, were identified. The accurate and reliable assignation of the carbon signals *3*, *6*, *7*, *8*, *15*, and *15′* was possible with 2D NMR (Figure S2C,D, as well as an explanation to this Figure).

**Figure 2:**
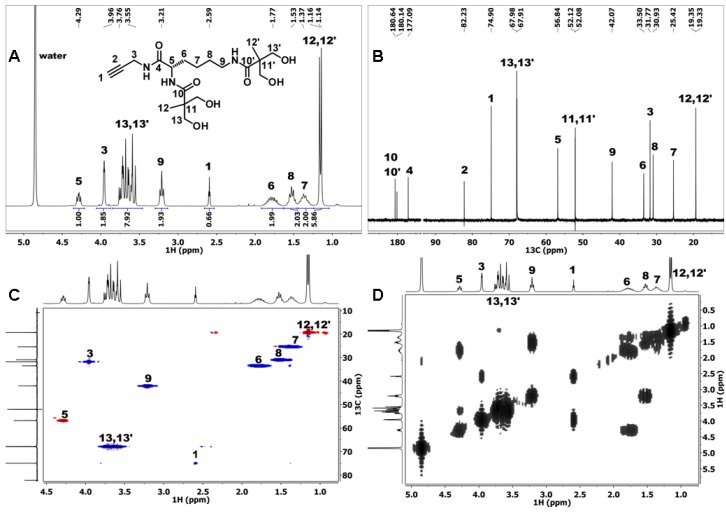
(**A**) ^1^H NMR; (**B**) ^13^C NMR; (**C**) gHSQCad NMR; and (**D**) COSY NMR spectra of dendron D recorded in D_2_O.

Mild acidic conditions (acetonitrile:TFA = 20:1) were applied to remove the acetonide protective group, which yielded the deprotected second-generation dendron D (Figure 1). 1D and 2D NMR were recorded to characterize the product D (Figure 2).

The ^1^H NMR spectrum of dendron D (Figure 2A) reveals the absence of the acetonide protective group signals, while the bis-MPA methyl groups’ signals (at 1.14 and 1.16 ppm) are still present. Dendron D was, thus, completely deprotected which was further confirmed by the absence of acetonide signals in ^13^C and gHSQCad NMR spectra (Figure 2B,C). The assignation of lysine proton signals *6*, *7*, and *8* was performed using a COSY NMR spectrum (Figure 2D), where signal *6* (1.77 ppm) was identified by its correlation with the proton *5* signal (4.29 ppm). The proton *7* signal (1.37 ppm) was then identified according to its COSY correlation with signal *6*. Finally, the signal *8* (1.53 ppm) was identified. The assignation of ^13^C NMR signals in the region between 25 and 34 ppm was done using the gHSQCad NMR spectrum where signals at 25.4, 30.9, 31.8, and 33.5 ppm were correlated to the proton signals *7*, *8*, *3*, and *6*, respectively (Figure 2C). In the gHSQCad NMR spectrum of the deprotected dendron D, the same anomaly was observed for acetylene CH group 1 as in the case of the protected precursor d-acetonide (Figure S2), [60]. 

### 3.2. Synthesis of Poly[l-Glutamate-co-(N-3-Azidopropyl)-l-Glutamine] (P(Glu-N_3_))

**Figure 3:**
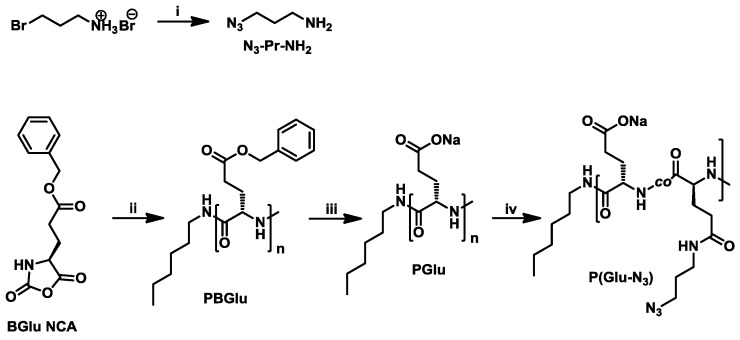
Reaction scheme for P(Glu-N_3_) preparation: (**i**) NaN_3_, water, 80 °C; (**ii**) *n*-hexylamine, DMF, 0 °C; and (**iii**) MSA, anisole, TFA, 0 °C then r.t.; (**iv**) N_3_-Pr-NH_2_, DMTMM, water, r.t.

P(Glu-N_3_) was prepared using a four-step synthetic approach (Figure 3, Supplemental Materials). N_3_-Pr-NH_2_ was prepared by substitution reaction using 3-bromopropane-1-amine hydrobromide and sodium azide [61,62]. BGlu NCA was prepared using triphosgene and was then polymerized by ROP using *n*-hexylamine as the initiator to prepare PBGlu. PBGlu was characterized by NMR, SEC-MALS, and MALDI-TOF MS to confirm the proposed structure and to determine the molar mass characteristics of the product (Figure S3).

The number-average degree of polymerization (DP_n_) was determined from ^1^H NMR spectrum (comparison of integrals of the *g* signal at 3.92 ppm for α-proton of BGlu moiety and the signal *a* at 0.82 ppm for methyl group of hexylamine initiator moiety, Figure S3A) as well as SEC-MALS chromatogram of PBGlu (Figure S3C), and was found to be 41 and 38, respectively. Additionally, SEC-MALS results revealed narrow molar mass distribution of PBGlu. (Figure S3A). The peak apex in PBGlu MALDI-TOF mass spectrum indicates the 36-mer. MALDI-TOF MS shows the presence of species with unmodified N-terminus and hexylamine C-terminus (signals A and C; potassium and sodium adducts) as well as the species with the pyrrolidone and hexylamine end groups (signal B; potassium adduct) (Figure S3D and Figure S4). The latter are formed by intramolecular cyclization of the amino end group with the adjacent benzyl ester.

PBGlu was then deprotected using acidic conditions (Figure 3) [63,64]. The polymer was dissolved in TFA and chilled in an ice bath. Anisole and MSA were then added. The reaction mixture was stirred at 0 °C for 20 min and then at room temperature for another 30 min. The product was precipitated using ice-cold diethyl ether and collected by centrifugation. PGlu was dissolved in NaHCO_3_ (sat.) and purified by dialysis against deionized water followed by freeze-drying. PGlu was characterized by NMR (Figure S5), MALDI-TOF MS (Figures S5 and S6), and SEC-MALS (Figure S7).

A DP_n_ value of 45 was determined for the PGlu sample from both ^1^H NMR spectrum (Figure S5A) and SEC-MALS data (Figure S7). SEC-MALS revealed narrow molar mass distribution (*Đ*_M_ 1.04). The MALDI-TOF mass spectrum shows the apex value *M*_apex_ at 5008.7 Da, corresponding to a PGlu species with 38 Glu units (Figure S5C). The most intensive set of signals in MALDI-TOF spectrum of the PGlu sample was assigned to the sodium adduct of a PGlu species with a hexylamine C-end and pyrrolidone N-end (Figure S5D, signal marked with D). The MALDI-TOF mass spectrum also revealed the presence of a species *D* which was identified as a monosodium salt of species D. Structures of the corresponding species identified in the PGlu MALDI-TOF MS spectrum are presented in Figure S6.

**Figure 4:**
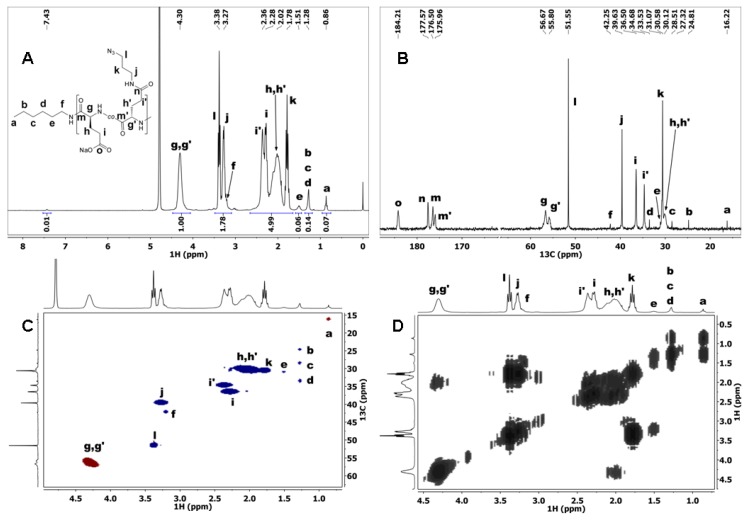
(**A**) ^1^H NMR; (**B**) ^13^C NMR; (**C**) gHSQCad NMR; and (**D**) COSY NMR spectra of P(Glu-N_3_) copolymer recorded in D_2_O.

P(Glu-N_3_) was then prepared by coupling N_3_-Pr-NH_2_ to PGlu in the presence of DMTMM reagent (Figure 3). The degree of substitution was controlled by the amount of the DMTMM reagent added to the reaction mixture (0.5 equivalent DMTMM per PGlu carboxylic group for DS 50% targeted value). N_3_-Pr-NH_2_ and PGlu were dissolved in deionized water and the mixture was neutralized with 1M HCl. DMTMM was then added and the reaction mixture was stirred for 24 h. Product, P(Glu-N_3_), was purified by dialysis and then freeze-dried. P(Glu-N_3_) was analyzed by 1D and 2D NMR experiments (Figure 4), SEC-MALS (Figure S7), and MALDI-TOF MS (Figure S8). The degree of substitution (DS) of the Glu units with N_3_-Pr-NH_2_ was determined from the ^1^H NMR spectrum (Figure 4A) where signals *j*, *l*, and *f* at ~3.3 ppm were integrated, and the signal *f* integral value (its value equals 2/3 of the signal *a* integral value) was subtracted. The presence of signal *f* was determined from the COSY and gHSQCad spectra of the P(Glu-N_3_) copolymer (Figure 4C,D). Signals *j* and *l* were assigned to the two CH_2_ groups of the N_3_-Pr-NH_2_ moiety; therefore their integral value was divided by four to determine the DS value of 43% which is close to the targeted 50% value. The DP_n_ value 44 for P(Glu-N_3_) was determined from its ^1^H NMR spectrum following the same procedure as for PBGlu and PGlu polypeptides. SEC-MALS (Figure S7) revealed a *M*_n_ value 8230 Da for P(Glu-N_3_), corresponding to DP_n_ 46 when using DS 43% from ^1^H NMR for the calculation. ^1^H NMR and SEC-MALS data are, thus, in very good agreement.

In the ^13^C NMR spectrum of the P(Glu-N_3_) copolymer a difference in chemical shift values between the unmodified and modified Glu carbon signals was between 0.5 and 7 ppm (Figure 4B). The largest difference was expectedly observed for the side chain carbonyl carbons *n* and *o*, followed by the adjacent carbon signals *i* and *i*’ which are separated by 2 ppm.

In the MALDI-TOF mass spectrum of P(Glu-N_3_) copolymer (Figure S8) an unresolved broad signal, due to instability of azide groups under MALDI conditions, was observed with *M*_apex_ 5600 Da, corresponding to P(Glu-N_3_) copolymer with DP_n_ of 33. The DP_n_ value, determined from the MALDI-TOF spectrum, is lower than the corresponding values determined form ^1^H NMR (44) and SEC-MALS (46) data. This disagreement was attributed to mass discrimination in the ionization/detection process. In general, the MALDI ionization/detection favors the low molecular weight species [65,66,67].

### 3.3. Synthesis of Dendronized Poly(l-Glutamate) (P(Glu-D))

**Figure 5:**
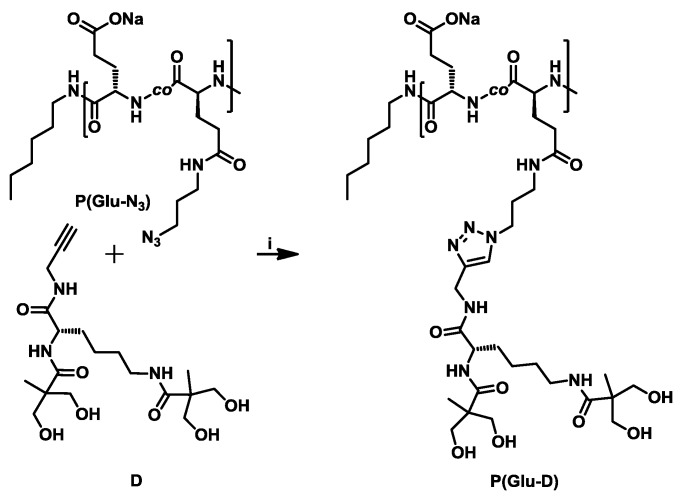
Reaction scheme for P(Glu-D) preparation. Reaction conditions: (i) CuSO_4_·5H_2_O, sodium (+)-ascorbate, water, r.t.

**Figure 6:**
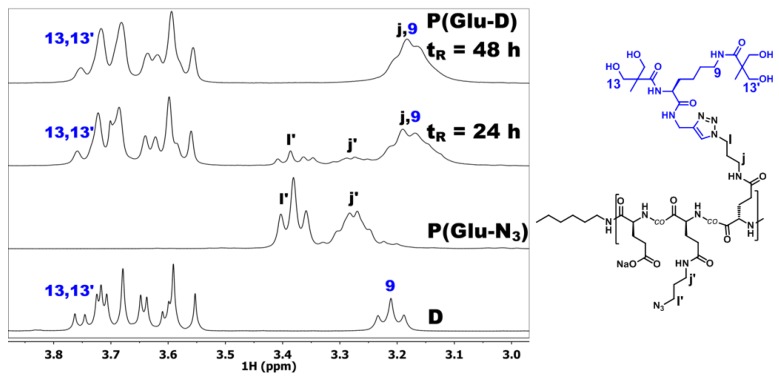
Monitoring the synthesis of P(Glu-D) with ^1^H NMR in D_2_O.

**Figure 7:**
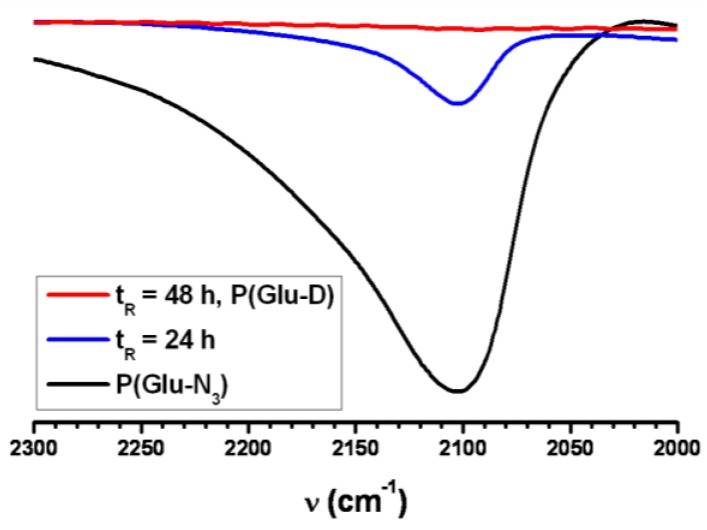
Monitoring the synthesis of P(Glu-D) with FT-IR. The region between 2000 and 2300 cm^−1^ is shown, where the azide band at 2100 cm^−1^ was monitored.

The dendronized copolymer P(Glu-D) was prepared by dendronization of the linear copolymer P(Glu-N_3_) with dendron D (Figure 5, Supplemental Materials). P(Glu-N_3_) contained pendant azide functional groups on 43% of the repeat units while the remaining unmodified Glu repeat units were in the form of sodium salt. The acetylene focal group was incorporated in the dendron D structure which can, thus, react with an azide-functionalized glutamate in the presence of Cu(I) catalyst to form a 1,4-disubstituted 1,2,3-triazole (Figure 5). The CuAAC reaction was monitored by ^1^H NMR (Figure 6) and FT-IR (Figure 7) by taking aliquots at 24 h and 48 h reaction times. The aliquots were dialyzed and freeze-dried before characterization.

In the ^1^H NMR spectrum of the aliquot taken at 24 h reaction time (Figure 6), an intensive multiplet between 3.5 and 3.8 ppm was observed and assigned to the bis-MPA moiety protons *13* and *13*’. Next, signals *l*’ and *j*’ were observed that were assigned to the non-dendronized Glu-N_3_ repeat unit signals. After 48 h reaction time the signals *l*’ and *j*’ were no longer present indicating completion of the dendronization reaction.

FT-IR spectra of the starting P(Glu-N_3_) copolymer and both aliquots were recorded to monitor the disappearance of the azide band at 2100 cm^−1^ (Figure 7). The FT-IR spectrum of the starting P(Glu-N_3_) reveals a very intensive band at 2100 cm^−1^, whereas the band was much less intense after 24 h reaction time. The FT-IR spectrum of the final aliquot at 48 h reaction time showed no presence of the azide band at 2100 cm^−1^, indicating that the azide functional groups had reacted quantitatively.

**Figure 8:**
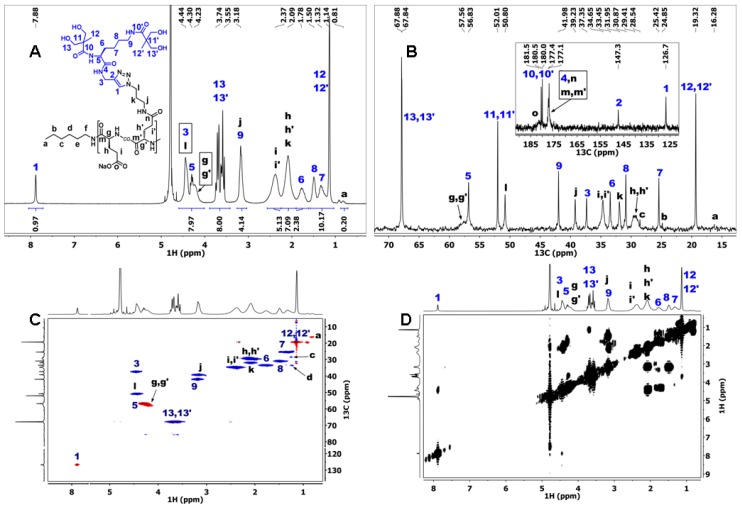
(**A**) ^1^H NMR; (**B**) ^13^C NMR; (**C**) gHSQCad NMR in (**D**) COSY NMR spectra of the P(Glu-D) dendronized copolymer in D_2_O.

**Figure 9:**
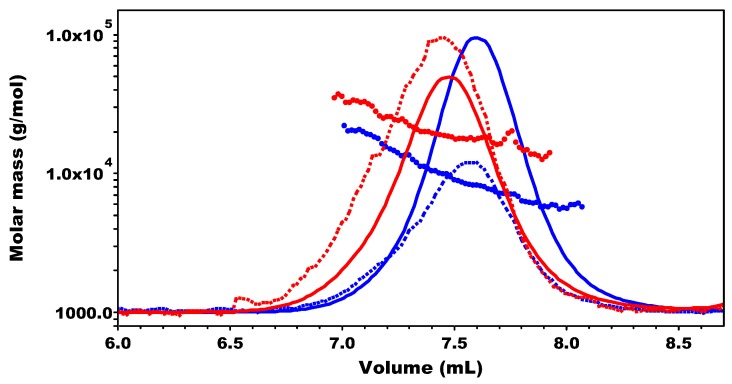
SEC-MALS chromatograms (solid lines: RI responses, dashed lines: LS responses at angle 90°) of P(Glu-N_3_) (blue) and P(Glu-D) (red) copolymers, together with molar mass *vs.* elution volume.

After 48 h of the dendronization reaction time the reaction mixture was successively dialyzed against EDTA solution, against 0.1 M NaCl, and against deionized water, followed by freeze-drying. The structure of the P(Glu-D) dendronized copolymer was investigated by 1D and 2D NMR experiments (Figure 8) while the molar mass averages and distribution were determined by SEC-MALS (Figure 9, Table 1).

The ^1^H NMR spectrum of the P(Glu-D)-dendronized copolymer was rather complex (Figure 8A). The signal *1* characteristic of the triazole proton was observed at 7.88 ppm. This signal indicated that the covalent triazole linkage was formed between the dendron’s acetylene focal group and the pendant azide groups of the P(Glu-N_3_) copolymer. Several signals were overlapping in the region between 4.2 and 4.5 ppm. In this region the signals *5*, *g*, and *g*’ were expected—however, the integral value indicates overlapping with additional signals. A multiplet between 3.5 and 3.8 ppm was assigned to the bis-MPA moiety CH_2_ signals *13* and *13*’. At 3.18 ppm, where the lysine proton *9* signal is expected, a signal was present but its integral value indicates additional overlapping. In the region below 2.4 ppm several signals were observed which were assigned to the protons *k*, the Glu protons *h* and *i*, the lysine protons *6*, *7*, and *8*, and the bis-MPA protons *12* and *12*’. A detailed analysis was done using additional NMR experiments.

In the gHSQCad spectrum of P(Glu-D) copolymer (Figure 8C) the proton signal at 4.44 ppm was correlated to two different CH_2_ carbons (*l* and *3*). The signals *l* and *3* were assigned from the COSY spectrum (Figure 8D) showing correlations to signals *k* and *1*, respectively. In the gHSQCad spectrum protons at 4.2–4.3 ppm were correlated to the CH carbons at ~57 ppm. In the ^13^C NMR spectrum of P(Glu-D) (Figure 8B) at 56–58 ppm a relatively sharp signal (*5*) is overlapped with a broad signal (*g* and *g*’). In the gHSQCad spectrum the proton signal at 3.18 ppm was correlated to two different CH_2_ carbons and the assignation was possible using the COSY spectrum where the signals *j* and *9* were correlated to the signals *k* and *8*, respectively.

In SEC-MALS an increase in the *M*_n_ value of P(Glu-D) (1.67 × 10^4^ Da) was observed when compared to the *M*_n_ value of the precursor P(Glu-N_3_) copolymer (8.23 × 10^3^ Da) (Figure 9, Table 1). The *M*_n_ value of P(Glu-D) determined by SEC-MALS was in perfect agreement with the theoretical *M*_n_ value indicating that coupling of dendron D to the linear copolymer P(Glu-N_3_) indeed proceeded quantitatively under very mild reaction conditions.

## 4. Conclusions

P(Glu-D), a dendronized copolymer (DP_n_: 46, DS: 43%, dendron generation: 2) was designed, synthesized, and characterized. It was prepared from a linear Glu-based copolypeptide and an l-lysine- and bis-MPA-based second-generation dendron. PGlu was modified using a bifunctional building block N_3_-Pr-NH_2_ and DMTMM coupling reagent under mild aqueous conditions and characterized by 1D and 2D NMR experiments, SEC-MALS, and MALDI-TOF MS. The dendron D, with an acetylene focal group, was then coupled to the pendant azide groups of the linear P(Glu-N_3_) copolymer to form triazole linkages. The dendron D was prepared by a multi-step synthetic approach. First, propargylamine was coupled to the succinimide-activated l-lysine carboxyl group. Then, bis-MPA-acetonide was coupled to the deprotected l-lysine amine groups in the presence of EDC and HOBt to prepare the acetonide-protected second-generation dendron with an acetylene focal group. The d-acetonide dendron was purified by flash-column chromatography, followed by removal of acetonide protection groups under mild acidic conditions. The deprotected second-generation dendron D with four peripheral hydroxyl groups was characterized by 1D and 2D NMR experiments. In the last step, dendron D was coupled successfully to the linear P(Glu-N_3_) copolypeptide under mild Cu(I) catalyzed conditions. The dendronization reaction was monitored by ^1^H NMR and FT-IR to show that the reaction was quantitative. The P(Glu-D)-dendronized copolymer was characterized by 1D and 2D NMR experiments and by SEC-MALS. The obtained data were in excellent agreement with the proposed structure and molar mass characteristics. Based on literature reports, we expect the newly-synthesized structure to be biocompatible, with potential for use in biomedical applications.

## Figures and Tables

**Table 1:** Molar mass data of P(Glu-N_3_) and P(Glu-D).

Sample	Calculated	SEC-MALS		
	*M*_n_(theor.) ^a^ (10^4^ Da)	*M*_n_ (10^4^ Da)	*M*_w_ (10^4^ Da)	*Đ*_M_
P(Glu-N_3_)	-	0.823	0.905	1.1
P(Glu-D)	1.65	1.67	2.00	1.2

^a^ Theoretical value *M*_n_(theor.) was calculated from (i) dendron D data: *M* = 415.48 g/mol) and (ii) P(Glu-N_3_) copolymer data: DS = 43%, determined from ^1^H NMR spectrum of P(Glu-N_3_), and DP_n_ = 46, determined from SEC-MALS chromatogram of P(Glu-N_3_).

## Supplementary Materials

The following are available online at www.mdpi.com/1996-1944/9/4/242/s1. Included in the supplementary materials are details regarding the individual steps towards the synthesis of dendronized poly(l-glutamate) and Figures S1–S8.
