# Supplementary material for: Synthesis of Dendronized Poly(l-Glutamate) via Azide-Alkyne Click Chemistry
Source: Materials (Basel). 2016 Mar 29;9(4):242. doi: 10.3390/ma9040242 (PMC5502894; doi:10.3390/ma9040242)
Supplement: Supplementary file 1 [file materials-09-00242-s001.pdf]

Materials **2016**, *9*, 242; doi:10.3390/ma9040242

Supplementary Materials: Synthesis of Dendronized Poly(L-Glutamate) via Azide-Alkyne Click Chemistry

Peter Perdih, Andrej Kržan and Ema Žagar

Synthesis of N_α,N_ε-di-Boc-L-Lysine Propargylamide (Boc-Lys(Boc)-P)

Boc-Lys(Boc)-OSu (5.12 g, 12 mmol, 1 equivalent) was dissolved in dichloromethane (120 mL). Propargylamine (777 μL, 12 mmol, 1.05 equivalents) and triethylamine (1.7 mL, 12 mmol, 1.05 equivalents) were added to the clear solution. The reaction mixture was stirred at room temperature for 20 h followed by washing with 1M $NaHSO_4$ (3 × 30 mL), 10% Na_2CO_3 (3 × 30 mL), and with brine (3 × 30 mL). The organic phase was dried over Na_2SO_4 and evaporated under reduced pressure. Yield: 3.86 g (87%).

Synthesis of L-Lysine-P Trifluoroacetate (Lys-P × 2TFA)

Boc-Lys(Boc)-P (3.82 g, 10 mmol) was dissolved in dichloromethane (40 mL), and then TFA (40 mL) was slowly added. The reaction mixture was stirred at room temperature for 75 min, and then the volatiles were evaporated under reduced pressure. The oily crude product was dissolved in acetonitrile (80 mL). Amberlyst® A21 (10 g) resin was added and the mixture was stirred at room temperature for 20 min. Afterwards, the resin was filtered and washed with acetonitrile. The filtrate was evaporated under reduced pressure. Yield: 2.86 g (70%).

Synthesis of Bis-MPA-Acetonide

Bis-MPA (16.5 g, 120 mmol) and PTSA monohydrate (0.23 g, 1.2 mmol) were suspended in acetone (90 mL). 2,2-Dimetoxypropane (22 mL, 180 mmol) was added to this mixture and stirred at room temperature for 3 h. Afterwards, 2 M ammonium in ethanol (0.6 mL, 1.2 mmol) was added to the clear reaction mixture, which was stirred for five more minutes. The reaction mixture was evaporated under reduced pressure and the solid residue was dissolved in dichloromethane (150 mL). Organic phase was washed with deionized water (3 × 20 mL), dried over Na_2SO_4, and evaporated under reduced pressure. Yield: 17.8 g (83%).

Synthesis of N_α,N_ε-di-(bis-MPA-Acetonide)-L-Lysine-P (D-Acetonide)

Lys-P × 2TFA (2.8 g, 13.8 mmol protonated amino groups) was dissolved with dichloromethane (100 mL) and triethylamine (5.9 mL, 42 mmol) was added. The reaction mixture was chilled in the ice bath, and then bis-MPA-acetonide (2.5 g, 14 mmol), HOBt (1.9 g, 14 mmol), and EDC (2.7 g, 14 mmol) were added. The reaction mixture was stirred for 20 h and allowed to warm up to room temperature. Then, the reaction mixture was washed with deionized water (2 × 30 mL), 1M $NaHSO_4$ (3 × 30 mL), 10% Na_2CO_3 (3 × 30 mL), and brine (3 × 30 mL). The organic phase was dried over Na_2SO_4 and evaporated under reduced pressure. The crude product was purified with flash-column chromatography (the product was eluted with ethyl acetate: hexane, 9:1, followed by pure ethyl acetate). Yield: 2.7 g (78%).

Synthesis of N_α,N_ε-di-(Bis-MPA)-L-Lysine (D)

D-acetonide (1.5 g, 3.0 mmol) was dissolved in acetonitrile (180 mL) and then TFA (9 mL) was added. The reaction mixture was stirred at room temperature for 30 min and then evaporated under reduced pressure. The oily product was dried overnight under vacuum, dissolved in deionized water (20 mL) and freeze-dried. Yield: 1.2 g (95%). IR: 3305, 2942, 2884, 1779, 1633, 1537, 1156, 1035, 700 cm^{-1}.

Synthesis of γ-Benzyl-L-Glutamate NCA (BGlu NCA)

A 250 mL flame-dried round-bottom flask was charged with BGlu (6.2 g, 26 mmol) and triphosgene (3.7 g, 13 mmol) under argon. Then, dry THF (130 mL) was added and the reaction mixture was stirred at 55 °C for 90 min. The clear reaction mixture was concentrated under vacuum followed by precipitation in hexane. The product was crystallized from THF/hexane three times. Yield: 6.1 g (89%).

Synthesis of Poly(γ-Benzyl-L-Glutamate) (PBGlu)

BGlu NCA (5.0 g, 19 mmol) was dissolved in dry DMF (62 mL) under argon in an ice-bath [1]. A solution of hexylamine (0.38 mmol) in dry DMF (1 mL) was added and the reaction mixture was stirred in an ice bath for two days. Then, the reaction mixture was precipitated into cold deionized water. The product was collected by centrifugation, washed with water several times, and freeze-dried to obtain white powdery material. Yield: 3.9 g (94%). SEC-MALS: M_n: 8.4 kDa, M_w: 8.5 kDa, $Đ_M$: 1.01. MALDI-TOF MS: M_{apex}: 8033 Da

Synthesis of Poly(L-Glutamate) (PGlu)

PBGlu (1.0 g, 4.6 mmol benzyl ester groups) was dissolved in TFA (11 mL) under argon and anisole (2.75 mL, 23 mmol) was added to the mixture. The mixture was chilled in an ice bath and then MSA (10.2 mL, 158 mmol) was slowly added. The reaction mixture was stirred in ice bath for 20 min followed by stirring for 30 min at room temperature. Afterwards, the crude product was precipitated using ice-cold diethyl ether and collected by centrifugation. The product was dissolved in saturated $NaHCO_3$, dialyzed (CE, 100–500 Da) against deionized water and finally freeze-dried to obtain PGlu as sodium salt. Yield: 0.6 g (86 %). SEC-MALS: M_n: 6.8 Da, M_w: 7.1 Da, $Đ_M$: 1.04. MALDI-TOF MS: M_{apex}: 5012 Da.

Synthesis of 3-Azidopropane-1-Amine

3-Bromopropane-1-amine hydrobromide (4.38 g, 20 mmol) was dissolved in deionized water (67 mL) and then NaN_3 (3.90 g, 60 mmol) was added. The reaction mixture was stirred at 80 °C for 20 h and, afterwards, it was concentrated under reduced pressure to one-third of the volume. The mixture was chilled in the ice bath and diethyl ether (50 mL) was added. Next, NaOH (2.40 g, 60 mmol) was slowly added while stirring. Under the clear organic phase an emulsion was formed which turned clear after addition of a small amount of brine (2 mL). The organic phase was separated and the water phase was washed with diethyl ether (3 × 20 mL). The combined organic phase was dried over Na_2SO_4 and diethyl ether was evaporated under reduced pressure. Yield: 1.5 g (75%).

Synthesis of Poly[L-Glutamate-co-(*N*-3-Azidopropyl)-L-Glutamine] (P(Glu-N$_3$))

A round-bottom flask was charged with 3-azidopropane-1-amine (0.36 g, 3.6 mmol), deionized water (23 mL) and PGlu (0.36 g, 2.4 mmol carboxyl groups). 1M HCl (3.6 mL, 3.6 mmol) was added to the clear solution and afterwards also the DMTMM (0.33 g, 1.2 mmol). The reaction mixture was stirred at room temperature for 24 h. After that time, the mixture was dialyzed (CE, 100–500 Da) against 0.1 M NaCl and then against deionized water, and then finally freeze-dried. Yield: 0.28 g (33%). SEC-MALS: M_n: 8.2 Da, M_w: 9.1 Da, $Đ_M$: 1.10. MALDI-TOF MS: M_{apex}: 5600 Da.

Synthesis of Dendronized Poly(L-Glutamate) (P(Glu-D))

Dendron D (82 mg, 0.2 mmol) was dissolved in deionized water (8 mL) and P(Glu-N$_3$) (67 mg, 0.16 mmol) was added. $CuSO_4 \times 5H_2O$ (6 mg, 0.02 mmol) and sodium-(+)-L-ascorbate (15 mg, 0.07 mmol) were added to the reaction mixture, which was then stirred at room temperature for two days. The solution was then dialyzed (RC, 1000 Da) against 0.2 M EDTA, against 0.1 M NaCl, and finally against deionized water. The resultant solution was freeze-dried. Yield: 55 mg (40%). SEC-MALS: M_n: 1.670 × 10^4 Da, M_w: 1.995 × 10^4 Da, $Đ_M$: 1.20. MALDI-TOF MS: M_{apex}: 11,500 Da.

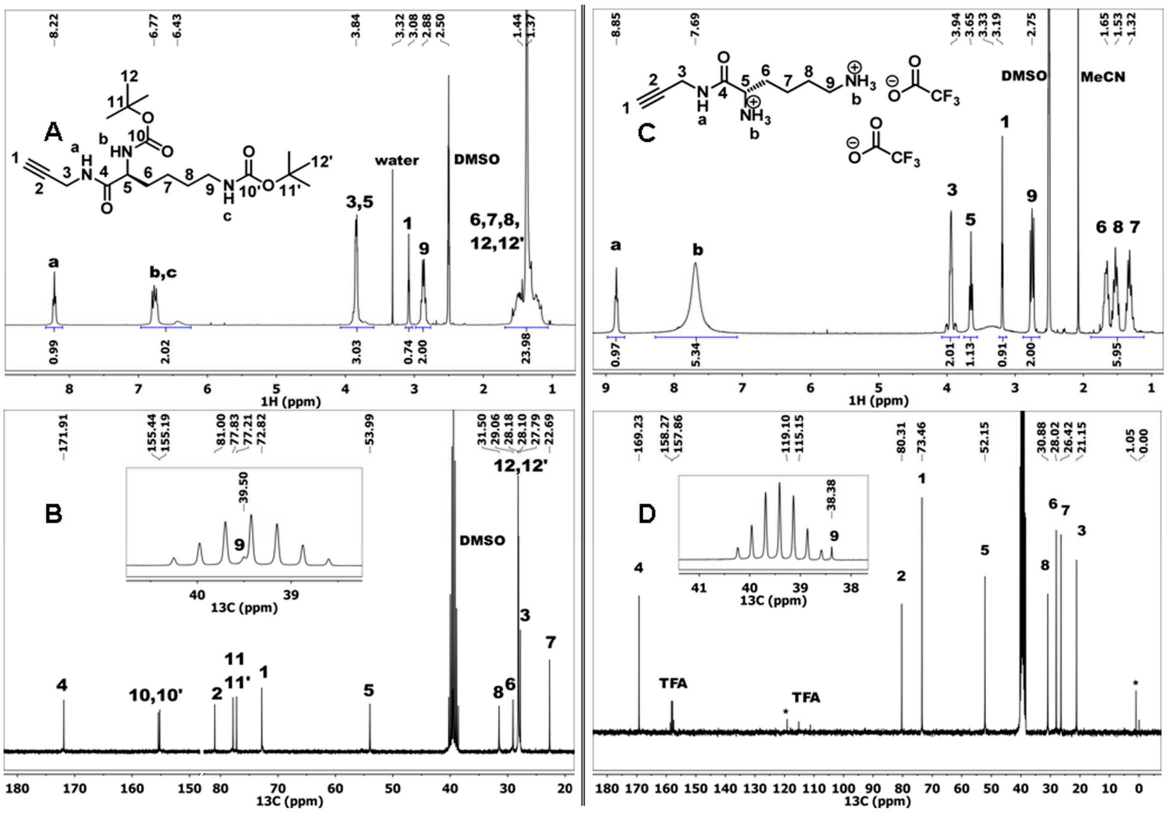

Figure S1. (**A**) ^{1}H NMR and (**B**) ^{13}C NMR spectrum of Boc-Lys(Boc)-P; (**C**) ^{1}H NMR; and (**D**) ^{13}C NMR spectrum of Lys-P × 2TFA. All the spectra were recorded in DMSO-d_6. The asterisks in spectrum D denote the residual acetonitrile signals.

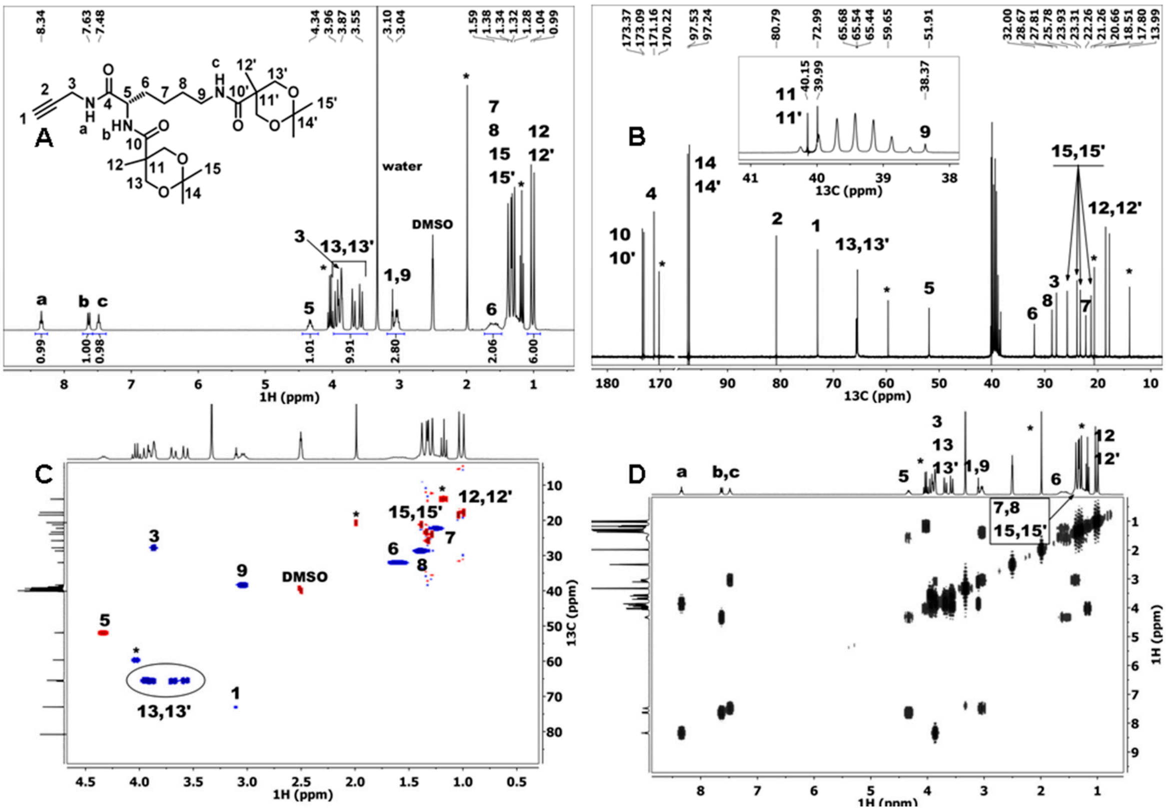

Figure S2. (**A**) ^{1}H NMR; (**B**) ^{13}C NMR; (**C**) gHSQCad NMR; and (**D**) COSY NMR spectra of D-acetonide recorded in DMSO-d_6. Asterisks denote the residual ethyl acetate signals.

Explanation to Figure S2: In the gHSQCad spectrum (2S, C) the integral values of the four CH_3 signals *15* and *15′* are, on one hand, positive (red color) while, on the other hand, the lysine CH_2 signals' *6*, *7* and *8* integral values are negative (blue color). Signals *6*, *7*, and *8* were assigned using 2D NMR spectra by the following procedure: doublet *b* (7.63 ppm), assigned to the amide proton signal in position N_α correlates with proton *5* (4.34 ppm) in the COSY spectrum (Figure S2D). Proton signal *5* also correlates with the signal at 1.62 ppm (signal *6*). According to the gHSQCad spectrum the proton signal at 1.62 ppm (signal *6*) is correlated to the carbon signal at 32.0 ppm. The signal of proton *6* (1.62 ppm) is, according to the COSY spectrum, correlated to the proton signal at 1.25 ppm, which was, thus, assigned to position *7*. The gHSQCad spectrum shows correlation between proton signal at 1.25 ppm and carbon signal at 22.3 ppm. The remaining CH_2 signal in the gHSQCad spectrum was assigned to the CH_2 group in position *8* and the assignation was confirmed by COSY, where correlation between signals *8* and *9* was observed.

Next, the carbon signal at 27.8 ppm was assigned to a CH_2 carbon according to the gHSQCad spectrum and it was observed that this carbon signal correlates to the proton signal at 3.87 ppm. COSY spectrum shows correlation between the amide proton *a* signal and the proton signal at 3.87 ppm which was, therefore, assigned to the position *3*. Furthermore, the proton signal *3* also correlates with the signal at 3.10 ppm, which was thus assigned to the acetylene proton *1*. In the gHSQCad spectrum an anomaly was observed for the signal *1*. For this signal, assigned to the acetylene CH group, the integral value was expected to be positive (red color), however, the spectrum shows a negative integral value. The reason for this anomaly lies in a very high one-bond C–H coupling constant value for the acetylene group (~250 Hz) [2]. The gHSQCad experiment was set for the coupling constant of 150 Hz, which agrees sufficiently with most C-H systems.

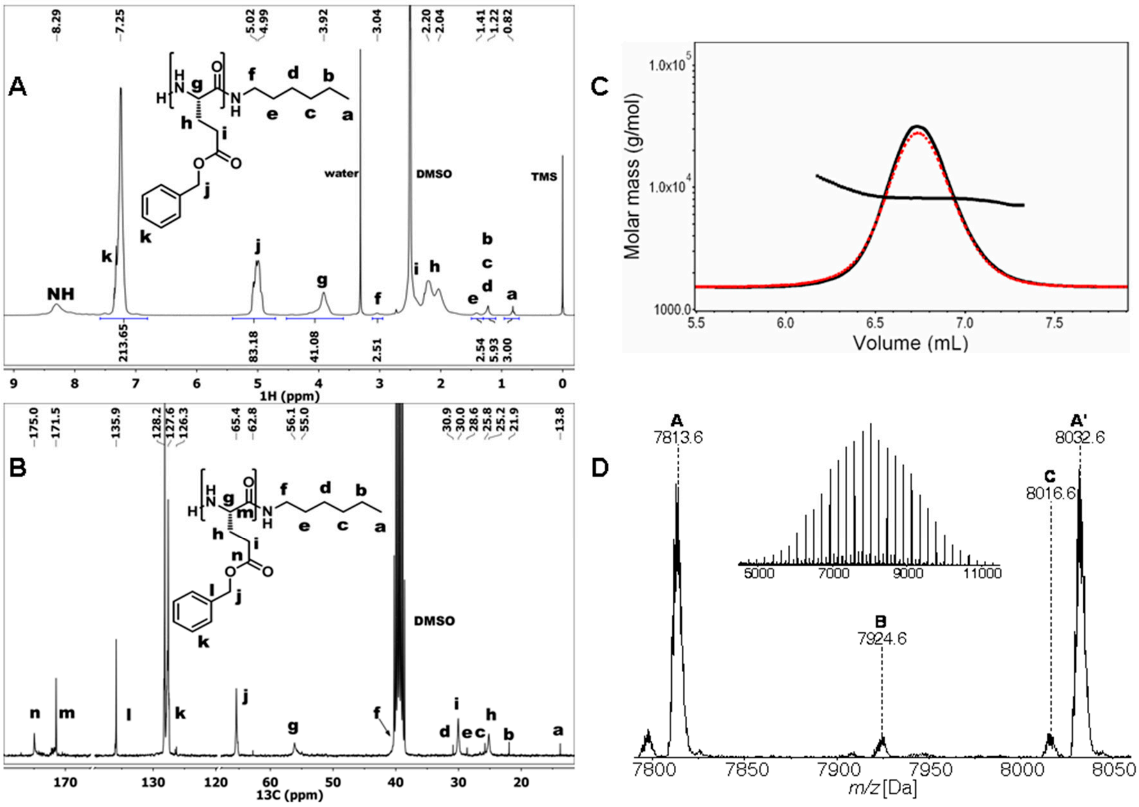

Figure S3. (**A**) ^1H NMR spectrum of PBGlu recorded in DMSO-d_6; (**B**) ^{13}C NMR spectrum of PBGlu recorded in DMSO-d_6; (**C**) Enlarged SEC-MALS chromatogram of PBGlu (solid line: RI response, dashed line: LS response at angle 90°) together with the molar mass vs. elution volume; and (**D**) Enlarged MALDI-TOF mass spectrum of PBGlu at the peak apex, recorded in reflector positive ion mode. Average molecular weights are annotated. Inset shows wider mass range of PBGlu mass spectrum.

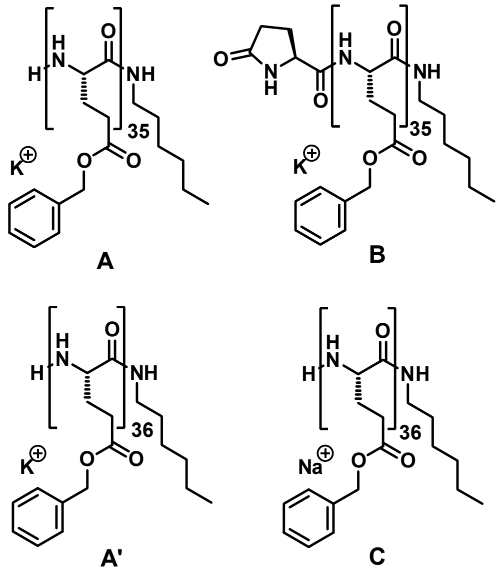

Figure S4. Structures of the species as assigned from the PBGlu MALDI-TOF mass spectrum (Figure S3D).

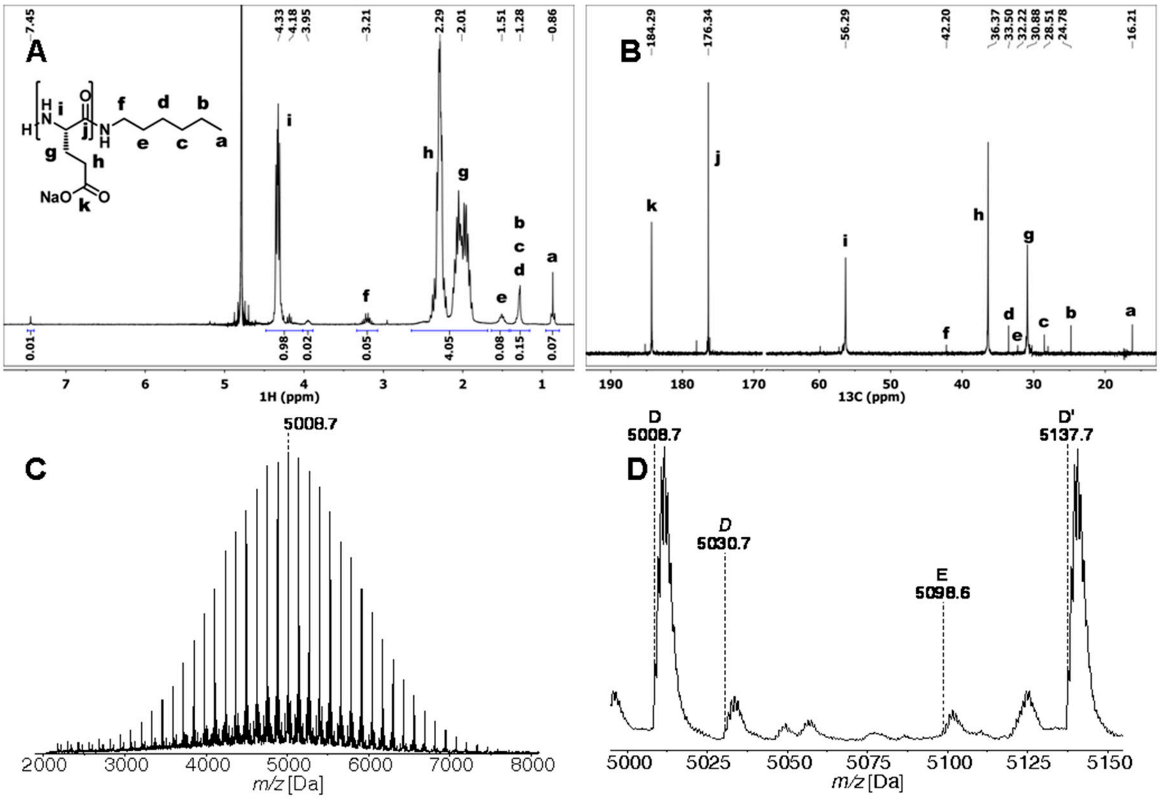

Figure S5. (**A**) ^{1}H NMR spectrum of PGlu recorded in D_2O; (**B**) ^{13}C NMR spectrum of PGlu recorded in D_2O; (**C**) MALDI-TOF mass spectrum of PGlu, recorded in reflector positive ion mode; and (**D**) enlarged MALDI-TOF mass spectrum of PGlu at the peak apex, recorded in reflector positive ion mode. Monoisotopic molar masses are annotated.

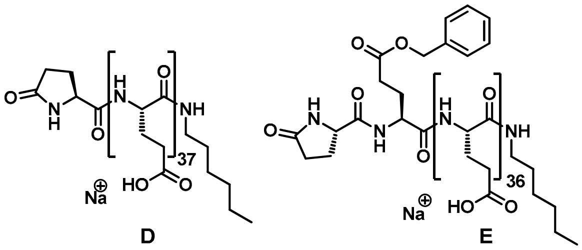

Figure S6. Structures of the species as assigned from the PGlu MALDI-TOF mass spectrum (Figure S5D).

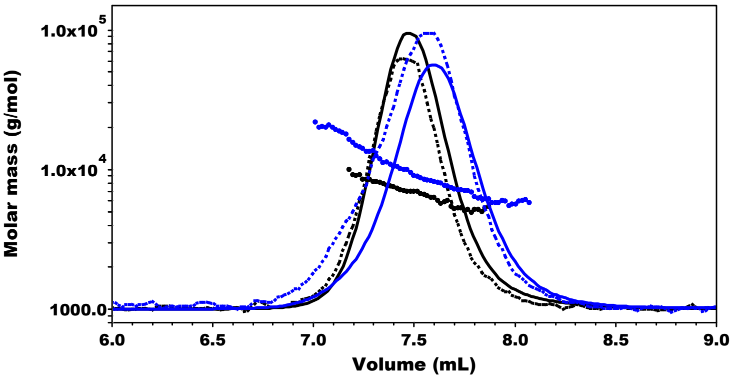

Figure S7. SEC-MALS chromatograms (solid line: RI response, dashed line: LS response at angle 90°) of PGlu (black) and P(Glu-N_3) (blue), together with the molar mass versus elution volume.

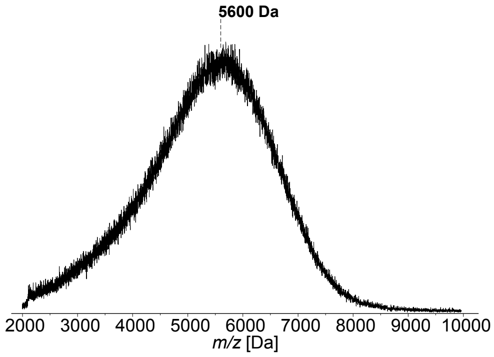

Figure S8. MALDI-TOF mass spectrum of P(Glu-N_3) copolymer recorded in reflector positive ion mode.

References

1. Habraken, G.J.M.; Peeters, M.; Dietz, C.H.J.T.; Koning, C.E.; Heise, A. How controlled and versatile is *N*-carboxy anhydride (NCA) polymerization at 0 °C? Effect of temperature on homo-, block- and graft (co)polymerization. *Polym. Chem.* **2010**, *1*, 514–524.
2. Claridge, T.D.W. *High-Resolution NMR Techniques in Organic Chemistry*; Newnes: Philadelphia, PA, USA, 2009.
